# Supporting Drug Development for Neglected Tropical Diseases Using Mathematical Modeling

**DOI:** 10.1093/cid/ciab350

**Published:** 2021-04-23

**Authors:** Martin Walker, Jonathan I D Hamley, Philip Milton, Frédéric Monnot, Sally Kinrade, Sabine Specht, Bélen Pedrique, Maria-Gloria Basáñez

**Affiliations:** 1 Department of Pathobiology and Population Sciences and London Centre for Neglected Tropical Disease Research, Royal Veterinary College, Hatfield, United Kingdom; 2 MRC Centre for Global Infectious Disease Analysis, Department of Infectious Disease Epidemiology and London Centre for Neglected Tropical Disease Research, Imperial College London, London, United Kingdom; 3 Drugs for Neglected Diseases initiative (DNDi), Geneva, Switzerland; 4 Medicines Development for Global Health, Melbourne, Victoria, Australia

**Keywords:** drug development, neglected tropical diseases, mathematical modeling, filariases, onchocerciasis

## Abstract

Drug-based interventions are at the heart of global efforts to reach elimination as a public health problem (trachoma, soil-transmitted helminthiases, schistosomiasis, lymphatic filariasis) or elimination of transmission (onchocerciasis) for 5 of the most prevalent neglected tropical diseases tackled via the World Health Organization preventive chemotherapy strategy. While for some of these diseases there is optimism that currently available drugs will be sufficient to achieve the proposed elimination goals, for others—particularly onchocerciasis—there is a growing consensus that novel therapeutic options will be needed. Since in this area no high return of investment is possible, minimizing wasted money and resources is essential. Here, we use illustrative results to show how mathematical modeling can guide the drug development pathway, yielding resource-saving and efficiency payoffs, from the refinement of target product profiles and intended context of use to the design of clinical trials.

## Teaser

Modeling is used in the commercial pharmaceutical sector to facilitate drug development. The efficiency payoffs of modeling in the resource-scarce neglected tropical disease (NTD) domain can be particularly valuable. Modeling should be routinely integrated into the NTD drug development pathway.

Mass drug administration (MDA), by which at-risk populations are regularly treated without the need for individual diagnosis, is at the core of the preventive chemotherapy strategy recommended by the World Health Organization (WHO) to eliminate 5 of the most globally prevalent neglected tropical diseases (NTDs): trachoma, soil-transmitted helminthiases (STHs), schistosomiasis, lymphatic filariasis, and onchocerciasis [1]. Four of these are earmarked for elimination as a public health problem (EPHP) in the new WHO 2030 NTD roadmap, while elimination of transmission (EOT) is proposed for onchocerciasis [2].

Therapeutic options for trachoma, the 3 major STHs (roundworm, whipworm, and hookworm), and schistosomiasis are reasonably efficacious and often curative, albeit with some exceptions, such as the inadequacy of benzimidazoles alone for treating whipworm [3]. While the therapeutic arsenal is limited for these NTDs—and therefore potentially vulnerable to emerging drug resistance—there is cautious optimism that if MDA is delivered at adequate frequency, coverage, and duration, then the WHO’s 2030 elimination goals can be met [4–6]. Similarly, although treatments for lymphatic filariasis are not generally considered “curative,” they are effective in strongly suppressing the microfilarial progeny (the stages that are transmitted to mosquito vectors) [7, 8] of adult filariae (macrofilariae) and may have partial anti-macrofilarial effects [9]. These treatments are therefore also considered compatible with EPHP [10].

The EOT goal for onchocerciasis places scrutiny on the efficacy of current treatment options for this filarial disease. A single standard dose (150 µg/kg) of ivermectin—the cornerstone of onchocerciasis MDA—is effective in mediating the killing of the microfilarial skin-dwelling transmission stages (to blackfly vectors) of adult *Onchocerca volvulus* and in inducing a temporary sterilizing (embryostatic) effect [11] (although suboptimal responses have been documented in Ghana [12]). But ivermectin only has partial killing or “curative” efficacy against adult worms and only after repeated MDA rounds [13]. Mathematical transmission dynamics modeling that integrates these pharmacodynamics indicates that ivermectin MDA alone, particularly if delivered annually, may be insufficient to achieve elimination in highly endemic areas where transmission is intense [14, 15]. In West Africa, before ivermectin MDA for onchocerciasis began in earnest in the late 1980s, transmission in 50–75% of endemic communities was deemed high (hyperendemic) or very high (holoendemic) [16]. Transmission remains ongoing in many such foci despite decades of intervention [17], attesting to the challenge of achieving widespread EOT by 2030.

The registration of moxidectin for the treatment of onchocerciasis in 2018 [18] was a significant milestone in enhancing the prospects of achieving EOT. Moxidectin, like ivermectin, is a macrocyclic lactone but with superior pharmacokinetics and pharmacodynamics, suppressing microfilariae to lower levels and for longer duration [18, 19]. This means that less transmission to vectors is possible between consecutive MDA rounds [18, 20]. But moxidectin will only be part of the solution to achieving widespread elimination of onchocerciasis. Financial and operational feasibility to allow uptake of moxidectin into MDA programs needs to be tackled. There are also safety concerns on the use of macrocyclic lactones in people heavily coinfected with *Loa loa* [21], another filarial parasite, that occurs in forest areas of central Africa [22]. Tetracycline antibiotics exert macrofilaricidal activity by depleting *Wolbachia* endosymbionts from *O. volvulus* and, crucially, are considered a safer alternative in people infected with *L. loa* (which lacks *Wolbachia*) [23, 24]. These represent important adjuvant or alternative therapies to the macrocyclic lactones and will likely become increasingly used if shorter treatment courses are found to be as efficacious as those currently available (eg, doxycycline, which requires 4–6 weeks of daily treatment) [25, 26].

For other NTDs, there have been similar recent developments in the therapeutic armory. The strong and sustained suppression of microfilaremia by the combination of ivermectin, diethylcarbamazine (DEC), and albendazole (so-called IDA therapy) presents a significant leap forward for the elimination of lymphatic filariasis [27, 28], albeit cautiously due to safety concerns of treating with DEC in Africa where many people are coinfected with onchocerciasis [28]. For whipworm, various combinations of existing antiparasitic drugs have shown improved efficacy over benzimidazoles alone [29, 30]. These examples all attest to drug development and repurposing being an active and important component of a holistic global strategy to enhance preventive chemotherapy for NTDs [14, 17, 31]. Continued drug development also provides insurance against the specter of emerging drug resistance, a particularly ominous prospect for diseases such as schistosomiasis, for which there is sole reliance on a single treatment [32].

Research and development (R&D) costs associated with drug development are high because of high attrition rates, with many promising molecules failing during preclinical development or in subsequent clinical safety and efficacy testing. The level of investment in R&D for new products for NTDs is nowhere near the level of funding required, and that funding, when available, is rarely allocated in a manner likely to ensure that products successfully move through the pipeline to reach end-users. It is therefore essential that stakeholders, funders, industry, academics, and nongovernmental organizations adopt a cooperative approach and share responsibility to reduce risks and overcome obstacles [33]. Joint efforts are being made to cut the cost of R&D for new drugs for NTDs and increase the attractiveness of this sector to funders and investors. For example, supportive programs by the US Food and Drug Administration (priority review voucher program) [34] and the European Medicines Agency (article 58) aim to increase incentives for companies to engage in NTD drug development.

Not-for-profit organizations (eg, Drugs for Neglected Diseases *initiative* [DND*i*], https://dndi.org; and Medicines Development for Global Health, https://www.medicinesdevelopment.com), supported through impact investment and philanthropic, governmental, and supranational grants and donations—have, over the last decade, started to fill the space left by commercial pharmaceutical companies who have little incentive to invest in new medicines without a profitable market [18, 35]. Indeed, the precedent that pharmaceuticals should be donated for NTDs may further disincentivize investment. For not-for-profit organizations—which naturally operate with fewer resources than their commercial counterparts—optimizing target product profiles (TPPs; see, eg, https://dndi.org/diseases/filaria-river-blindness/target-product-profile/) and the design of clinical trials [36] is essential to maximizing the efficiency of the drug development pathway. Modeling of all types has long been used in the commercial pharmaceutical sector to inform trial design [37]. However, it has only recently been adopted in the preclinical [38] and clinical [36] NTD domain, where resource-saving payoffs can result from better informed decision making and quantitative insights on proposed TPPs.

Since drug discovery and development for NTDs is largely driven by the unmet medical needs identified by the global health community and without the possibility of significant financial returns on investment, efficient use of money and other resources to deliver development programs is essential. To ensure that any development project is able to address the complex requirements of patients and healthcare providers with drug and formulation characteristics suitable for storage and use in tropical climates, the TPP provides a carefully defined framework for describing both the ideal and acceptable attributes of a novel medicine. In particular, the TPP must define the necessary characteristics and associated minimum and desired criteria against which a new therapy will be assessed. This may include relative safety, efficacy, effectiveness, and superiority against existing treatments. For NTDs in the era of elimination, specific challenges need to be tackled, beginning with the absence of a healthy drug discovery process. Further, the TPP should consider not only the individual-level therapeutic benefits but also the capacity for a new drug to facilitate elimination at the population level. But challenges can arise when properties conferring individual-level therapeutic benefits do not completely align with those most compatible with enhancing elimination.

**Figure 1.**
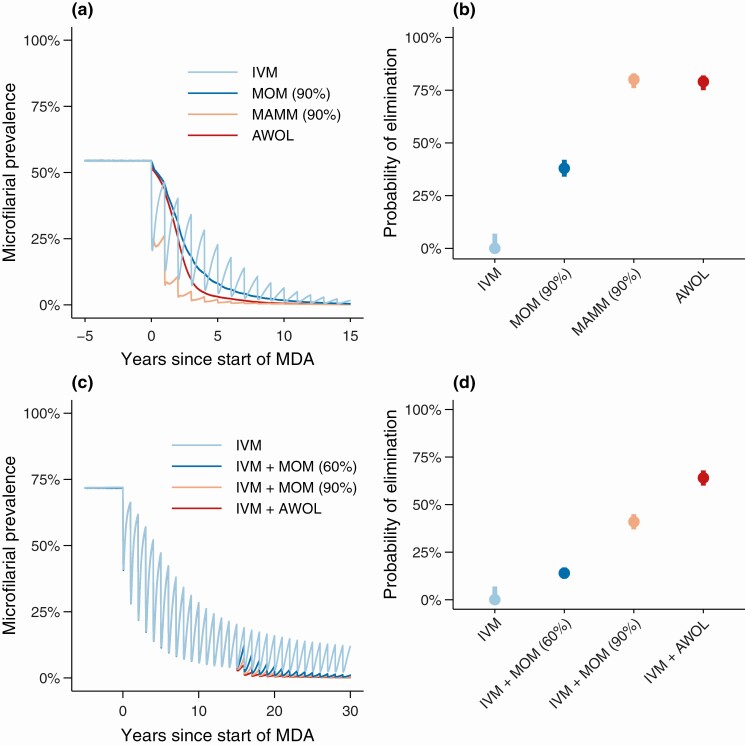
Projected dynamics, using EPIONCHO-IBM [39], of *Onchocerca volvulus* microfilarial prevalence. Panel *a* shows dynamics through 15 years of annual MDA with IVM or using a macrofilaricidal therapy in a mesoendemic setting. The light-blue line indicates MDA with IVM. Blue, orange, and red lines indicate, respectively, MDA with a hypothetical macrofilaricidal-only macrofilaricide (MOM) with 90% efficacy (90% of adult worms killed within 3 months following treatment) [36], a hypothetical macrofilaricidal and microfilaricidal macrofilaricide (MAMM; ie, a macrofilaricide with additional microfilaricidal activity) with 90% efficacy, and an anti-*Wolbachia* (AWOL) therapy (with 90% efficacy and pharmacodynamics based on those of doxycycline and prophylactic effect against incoming worms during the treatment course) [26]. The (model-predicted) probabilities of elimination (95% confidence intervals as error bars) for this scenario are depicted in panel *b*: 0% (0–7%), 38% (34–42%), 80% (76–83%), and 79% (75–82%) for IVM, MOM (90%), MAMM (90%), and AWOL, respectively. Panel *c* shows dynamics through 15 years of MDA with IVM followed by 15 years of IVM alone or in combination with a macrofilaricidal therapy in a hyperendemic setting. The light-blue line indicates MDA with IVM. Blue, orange, and red lines indicate, respectively, MDA with IVM for 15 years followed by the addition of either a MOM (with 90% or 60% efficacy, respectively) or an AWOL therapy. The (model-predicted) probabilities of elimination (95% confidence intervals) in this scenario are shown in panel *d*: 0% (0–7%), 14% (11–17%), 41% (37–45%), and 64% (60–68%) for IVM, IVM + MOM (60%), IVM + MOM (90%), and IVM + AWOL, respectively. Model projections assumed a hypothetical treatment coverage of 80% with 1% systematic nonadherence. Potential differences in eligibility criteria for different drugs (which would affect treatment coverage) are not considered in the interests of showing a simple vis-à-vis comparison of the impact of different pharmacodynamic properties. Abbreviations: IVM, ivermectin; MDA, mass drug administration.

We can illustrate this conundrum by considering the (individual-level) efficacy and (population-level) effectiveness of macrofilaricides (drugs that kill adult filariae) for the treatment of onchocerciasis. Figure 1a shows the modeled dynamics (using the transmission model EPIONCHO-IBM) [39] of microfilarial prevalence elicited by 4 drugs—with distinct pharmacodynamic properties—given annually by MDA in an onchocerciasis-endemic community. The strong microfilaricidal properties of ivermectin elicit immediate but transient declines in prevalence, which in the long term can suppress transmission to the point of elimination (although this is not necessarily the case when simulating more highly endemic populations) [39]. By contrast, macrofilaricides with either direct killing activity (MOM/MAMM in Figure 1a) or indirect depletion of endosymbiotic *Wolbachia* [26] (AWOL in Figure 1a)—but without substantial microfilaricidal action—elicit only slow reductions in microfilaridermia but can, in the long term, enhance the likelihood of elimination. Therefore, from the population perspective, a TPP may stress the importance of developing a macrofilaricide or long-term sterilizing drug with efficacy that increases the likelihood (probability) of elimination compared with ivermectin. But, from an individual perspective, as microfilariae are associated with the majority of clinical manifestations [40, 41], the slow decline in microfilaridermia elicited by a macrofilaricide without significant microfilaricidal activity may be associated with unacceptably slow therapeutic benefit.

The examples shown in Figure 1a are deliberately simple to enable vis-à-vis comparison of the effectiveness of drugs with contrasting pharmacodynamics. In reality, it is unlikely that a new macrofilaricide would be used as a replacement to ivermectin. It would more likely be used either to complement ivermectin in epidemiological settings where elimination has proved elusive and requires acceleration or in difficult-to-treat areas (such as where *L. loa* is highly co-endemic) as part of focused MDA or test-and-treat or not-treat strategies. As an example, Figure 1b illustrates the complementary use of a macrofilaricide with ivermectin in settings with a long (15-year) history of annual MDA intervention. Particularly noteworthy here is that the addition of a macrofilaricide—even with a modest 60% efficacy (assuming 60% of adult worms are killed within 3 months of administration)—results in an increased probability of elimination compared with ongoing ivermectin treatment alone. This example encapsulates the challenge of designing TPPs when faced with competing individual- versus population-level properties. We know from clinical trial simulation that demonstrating the benefit of a macrofilaricide with modest efficacy would likely require prohibitively long follow-up times and/or very large sample sizes [36]. Yet, should a macrofilaricide with 60% efficacy be discarded on this basis when it may be useful in accelerating progress towards elimination in mature intervention programs?

With these insights, one may choose to focus from the outset on comparing against ivermectin, combination therapies with pharmacodynamics similar to those of a a macrofilaricide with microfilaricidal action as shown in Figure 1a. This would restrict regulatory-approved usage to a combination therapy, although use of the macrofilaricide as monotherapy or in alternative combinations could continue to be further explored in clinical trials. Clinical studies supporting such combination use would be restricted to settings that align closely with the intended use scenario (eg, mature MDA programs where elimination has proved elusive and requires a new strategy). However, determining patients' past use of ivermectin is highly challenging and interpreting trial outcomes is complicated by the drug’s cumulative pharmacodynamics on *O. volvulus*, which accrue with long-term use [13]. Irrespective of the chosen path, transmission dynamics modeling can assist with evaluating the likely effectiveness of a new drug or combination in its proposed context of use and with practical decision making to optimize the design of trials to demonstrate efficacy. Indeed, without modeling, the implications of desired pharmacodynamic properties defined in the TPP may not be fully appreciated.

The use of mathematical modeling to support decision making along the NTD drug development pathway is still in its infancy. Pharmacokinetic-pharmacodynamics modeling has been used to translate preclinical data to humans [38] and transmission modeling has been used to make projections on the long-term epidemiological impact (effectiveness) of new drugs (eg, moxidectin for onchocerciasis) [18, 20] or repurposed combinations (eg, IDA for lymphatic filariasis [27] or benzimidazoles plus ivermectin for whipworm [42]) that have already been demonstrated as safe and efficacious in clinical studies. In onchocerciasis, we have partnered with DND*i* to use transmission modeling at earlier stages of clinical development to inform the design of trials [36], but this is yet to become more commonplace for other NTD drug development programs.

Naturally, the usefulness of models is defined by the quality of the underlying assumptions and, as in any practical application, it is crucial to communicate uncertainty in a clear and transparent manner [43]. In supporting drug development, it is essential that modeling acknowledges that the precise pharmacodynamics of a new drug (or indeed an existing comparator) will not be completely understood from the outset and exploration of the impact of key assumptions is paramount. Notwithstanding, qualitative insights remain useful and can help guide design decisions aimed at resolving uncertainties. For example, modeling may indicate (within a range) a minimum macrofilaricidal efficacy below which a new drug would not provide sufficient acceleration towards elimination. Armed with this insight, a clinical trial can be designed to collect data at particular time points that may not be most appropriate for final outcome assessment but will provide early information on likely pharmacodynamics properties (eg, early rates of microfilarial depletion) [36].

The illustrative results presented here aim to show the utility of transmission models when used in a prospective capacity to support decision making along the NTD drug development pathway. Specifically, modeling can help inform TPPs by projecting the likely effectiveness of new drugs, with different proposed pharmacodynamics, used in different intervention and epidemiological contexts. Modeling can also help to identify particular pharmacodynamic properties that may be useful for enhancing elimination—but not necessarily of immediate therapeutic benefit to the individual—and guide the appropriate design of phase 2/3 efficacy trials [36]. The integration of transmission modeling with economic cost-benefit analyses is a further important tool that remains relatively underused in the development of treatments for NTDs [44] but is a crucial component for informing donor investment decisions. Overall, we believe that mathematical transmission modeling should be more routinely adopted to inform NTD drug development decisions.

## Notes

***Author contributions.*** M. W. drafted the manuscript; J. I. D. H. performed the modeling; all authors commented for intellectual input and edited and approved the final version.

***Acknowledgments.*** The authors are indebted to Rebecca Smith, PhD, for her comments and feedback on the final submitted version of the manuscript.

***Financial support.*** M. W. and M.-G. B. acknowledge financial support from the Drugs for Neglected Diseases *initiative* (DND*i*; https://www.dndi.org). M. W., J. I. D. H., and M.-G. B. also acknowledge funding from the NTD Modelling Consortium by the Bill and Melinda Gates Foundation (grant number OPP 1184344). M.-G. B. further acknowledges joint center funding (grant number MR/R015600/1) by the Medical Research Council (MRC) and the UK Department for International Development (DFID) under the MRC/DFID Concordat agreement, which is also part of the European and Developing Countries Partnership for Clinical Trials (EDCTP2) program supported by the European Union. P. M. acknowledges support by a Doctoral Training Program studentship of the UK MRC.

***Potential conflicts of interest.*** F. M., S. S., and B. P. work at the organization (DND*i*) that partly sponsored this study. However, the study sponsor did not have any influence in the study design, analysis, interpretation of results, the writing of the manuscript, or the decision to submit the paper for publication, other than critical, independent, and scientific contribution. No payment was received from any other funding sources or pharmaceutical company. The corresponding author had final responsibility for decision to submit for publication. All other authors report no potential conflicts. All authors have submitted the ICMJE Form for Disclosure of Potential Conflicts of Interest. Conflicts that the editors consider relevant to the content of the manuscript have been disclosed.
